# Complete genome sequence of *Pseudomonas entomophila* strain TVIN-A01

**DOI:** 10.1128/MRA.00977-23

**Published:** 2024-01-18

**Authors:** Triveni Shelke, Nirmal Singh Mahar, Vanika Gupta

**Affiliations:** 1 Department of Biochemical Engineering and Biotechnology, Indian Institute of Technology, New Delhi, India; 2 Department of Zoology, Delhi University, Delhi, India; The University of Arizona, Tucson, Arizona, USA

**Keywords:** nanopore, long read, *Pseudomonas*, hybrid assembly

## Abstract

The complete genome sequence of *Pseudomonas entomophila* strain TVIN-A01 is reported in this manuscript. It is a commonly used laboratory strain of *Pseudomonas entomophila* known to infect insects and, hence, often studied in host-pathogen interactions. Oxford Nanopore sequencing and Illumina sequencing were performed to assemble the genome completely.

## ANNOUNCEMENT

*Pseudomonas entomophila* is a gram-negative, rod-shaped bacteria that resides in soil and water (1). It is used to study insect immunology due to its pathogenicity to insects like *Drosophila melanogaster*. It encodes insecticidal toxins, peptides, and several secondary metabolites against the host insect (1). These properties make the bacteria a suitable model for evaluating insect host-pathogen interactions (2).

*Pseudomonas entomophila* strain L48 was obtained from Prof. Bruno Lemaitre’s lab and isolated from *Drosophila melanogaster* (1). The bacterium was streaked on Luria Bertani (LB) agar plate and incubated at 27°C overnight. A single colony was picked, and the bacterium was cultured overnight in LB broth at 27°C, 160 rpm. Glycerol stocks were prepared from this culture. The glycerol stocks were revived by streaking the bacteria on LB agar plate and incubating at 30°C overnight. A single colony from the bacterial plate was inoculated in Luria Bertani broth and incubated overnight at 30°C. A pellet was obtained from the grown bacterial culture when centrifuged at 4,000 rpm for 10 minutes. The resultant pellet was then diluted with 1 mL Phosphate Buffered Saline (PBS). Genomic DNA was isolated from 250 μL of diluted pellet culture using a Zymobiomics DNA miniprep kit (Zymo Research, Irvine, CA, USA) (D4300) as per the manufacturer’s instruction. The unsheared or unfragmented genomic DNA was then sequenced using MinION Mk1B sequencer (Oxford Nanopore, UK) with R9.4.1 chemistry and library protocol SQK-LSK109 (Oxford Nanopore Technologies, Gosling Building, Oxford Science Park, Edmund Halley Road, Oxford OX4 4DQ, UK). The raw sequencing files were basecalled using Guppy basecaller version 6.3.8 with high accuracy (HAC) model. And 180,000 reads were obtained with an average read length of 3.077 kb, and adapter sequences trimmed when basecalling using Guppy basecaller. The short-read Illumina NovaSeq sequencing was performed on the same isolated genomic DNA by RedCliffe Lifesciences Pvt. Ltd (Sector-63, Electronics City Center, Noida 201301, UP, India), and approximately 20 million paired-end reads were obtained. The Nanopore sequencing reads were error-corrected using the Illumina sequencing reads by FMLRC2 version 0.1.7 (3) and ropeBWT2-r187 (4). The corrected Nanopore reads were then *de novo* assembled using Flye version 2.9.2 (5), resulting in three contigs. The contigs were scaffolded using the Patch module of RagTag version 2.1.0 (6) with *Pseudomonas entomophila* L48 genome (GCA000026105) as the reference. Polishing of the scaffold was done using long-read data by Medaka version 1.8.0 (7) and short-read data by Polypolish version 0.5 (8) for one and four rounds, respectively. The final assembly was 5,888,183 bp long with 64.16% Gaunine Cytosine (GC) content, as evaluated by QUAST version 5.0.2 (9).

**Fig 1:**
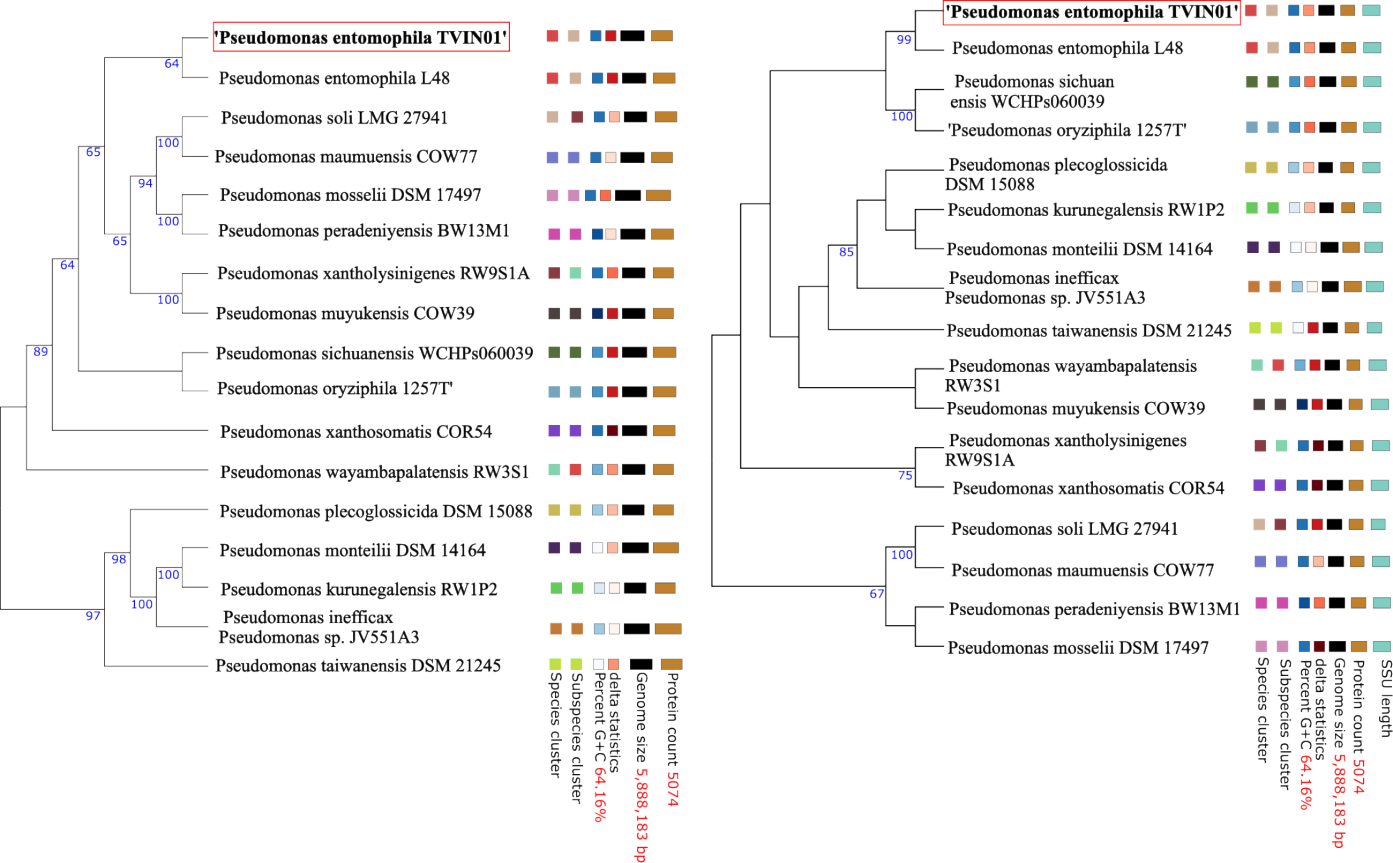
Phylogenetic tree based on the whole genome sequence that shows species cluster, sub-species cluster, percent G + C, delta statistics, the genome size (in bp), protein count, and ssu length (in bp). The numbers above branches are Genome BLAST Distance Phylogeny (GBDP) pseudo-bootstrap support values from 100 replications. (**A**) Whole genome sequence-based phylogenetic tree confirming the species *Pseudomonas entomophila*. (**B**) Phylogenetic tree based on the 16S ribosomal gene generated using TYGS server.

Furthermore, we used Benchmarking Universal Single-Copy Orthologs (BUSCO) version 5.2.2 (10) with the bacteria_odb10 database to assess the completeness of the final scaffolded assembly. Of the 124 orthologs searched in the database, 123 were complete and single-copy (99.2%), whereas one was fragmented (shorter than expected). To confirm the genome species, the 16S rDNA sequence was extracted using Barrnap version 0.9 (11) and aligned against the 16S ribosomal DNA sequences database using BLASTn web interface (12). The 16S rDNA gene showed 100% query coverage and 100% sequence similarity with *Pseudomonas entomophila* L48. Furthermore, the phylogenetic analysis based on 16S rDNA as well as the whole genome sequence was done using Type (Strain) Genome Server (TYGS) (13) software, which uses distances calculated from the Genome Blast Distance Phylogeny method which also confirmed *Pseudomonas entomophila* as the species of interest (Fig. 1).

Finally, genome annotation was done using PGAP version 6.6 (14), which annotated 5,215 genes, including 5,111 coding sequences (CDS), 22 rRNAs, 78 tRNAs, and 27 pseudogenes. Default parameters were used for all software unless otherwise specified.

## Data Availability

This Whole Genome Shotgun Project has been deposited at DDBJ/ENA/GenBank under the accession number GCF_031312015.1 and the Bioproject PRJNA1004059. Raw reads are accessible via NCBI with the accession numbers SRR25919843 and SRR25919842. The bacterial assemblies used for making the phylogenetic trees are GCF_000026105.1, GCF_900110655.1, GCF_019139675.1, GCF_000621225.1, GCF_014268935.2, GCF_014268885.2, GCF_019139535.1, GCF_003231305.1, GCF_003940825.1, GCF_019139835.1, GCA_014268975.2, GCF_000688275.1, GCF_000621245.1, GCF_014269245.2, GCF_900277125.1, GCF_000425785.1.
